# Microwave ablation of splenic cyst: A case report

**DOI:** 10.1016/j.amsu.2019.03.014

**Published:** 2019-04-12

**Authors:** Radmila Karpova, Artem Mishin, Samir Guseinov

**Affiliations:** a Department of Faculty Surgery №1 of I.M. Sechenov, First Moscow State Medical University (Sechenov University), Russia; b International School "Medicine of Future" of Biomedical Park of I.M. Sechenov, First Moscow State Medical University (Sechenov University), Russia; c Medical Department of I.M. Sechenov, First Moscow State Medical University (Sechenov University), Russia

**Keywords:** Microwave ablation, Splenic cyst, Mini-invasive surgery

## Abstract

**Introduction:**

Non-parasitic spleen cyst (NSC) is a relatively rare and difficult to diagnose disease, which rapture due to a traumatic impact to the spleen, can lead to the peritonitis.

**Case presentation:**

We present the case of a 30-year-old woman with a 7.5 × 7.5 × 5 cm NSC, who underwent the microwave ablation (MWA) of the splenic cyst.

**Results:**

The procedure was performed under intravenous anesthesia. MWA of the cysts was performed using a probe placed in the cavity of the cyst, with the frequency of 902–928 MHz delivered during 15 minutes. The postoperative period was uneventful, and the patient was discharged after 2 days.

**Conclusion:**

We demonstrated that MWA can be utilized as a novel, minimally-invasive, and cost-effective approach in NSC treatment.

## Highlights

• Case report of a 30-year-old female patient presented with non-parasitic spleen cyst.
• Microwave ablation is a promising approach in non-parasitic spleen cyst treatment.
• Microwave ablation is an alternative for splenectomy.

## 1 Introduction

Non-parasitic splenic cyst (NSC) is a relatively rare disease which is hard to diagnose. Clinical manifestations of NSC depend on the volume of the liquid inside the cyst, and possible complications, such as hemorrhage or abscess [1]. The most dangerous consequence of NSC is its rupture due to, for instance, a traumatic impact to the spleen, leading to development of peritonitis [2].

Many authors prefer splenectomy as the most radical method of treating NSC, especially for large cysts [3]. With the introduction into clinical practice of minimally invasive treatment methods under the control of ultrasound and fluoroscopy X-ray, most surgeons perform organ-preserving operations during NSC, which significantly improves the patient's quality of life [4]. At the same time, sclerotherapy and embolization of the NSC are expensive approaches, with the number of relapses up to 12.5%, and the average bed-day of 18 ± 0.5 [5,6].

The method of microwave ablation (MWA) was successfully utilized in the treatment of neoformations in parenchymal organs, including liver hemangiomas. The present case demonstrates the successful use of MWA in the treatment of NSC under the control of ultrasound and fluoroscopy X-ray.

## 2 Case presentation

On May 29, 2016, the patient – a woman of 30 years old, was accepted to the Sechenov University Clinical Hospital No. 1 with occasional pulling pain in left hypochondrium, nausea, and low-grade fever. The examination revealed a cyst in the spleen. The reaction of Kasoni and ELISA for Human Cystic echinococcosis was negative. The diagnosis of NSC was established. On June 13, 2016, the patient was admitted to the Department of Surgery for the surgery. A percutaneous puncture and MWA of the splenic cyst were performed under the control of ultrasound and X-ray fluoroscopy.

At admission: The spleen is of 20 × 10 cm, with the lower pole being palpated in the area of the mesogastrium, of dense consistency, and painless during palpation.

During examination: The blood and urine test results were within normal ranges, except for the erythrocyte sedimentation rate (ESR), which was 29 mm/h. Thrombocytopenia was not detected, with the values of platelets of 315 thousand/μl.

**Fig. 1:**
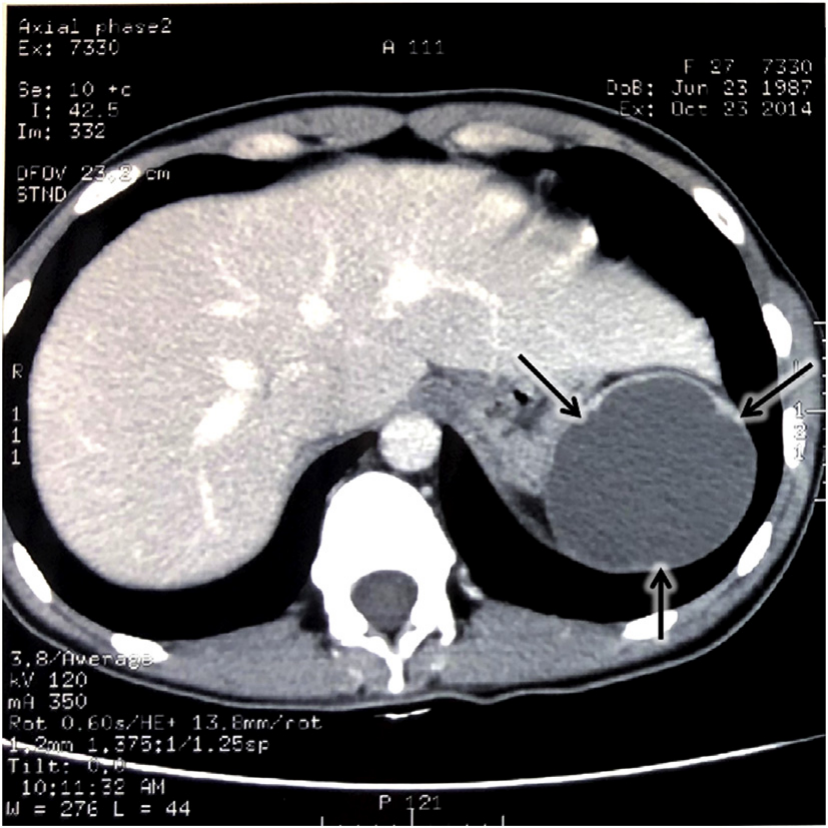
Pre-surgery CT scan of the abdominal region performed on October 23, 2014. Arrows indicate the splenic cyst of 7.5 × 7.5 × 5 cm.

Ultrasonography and computer tomography (CT) of abdominal organs: The spleen is of 20 × 10 cm, with clear, even contours, and homogeneous structure. In the upper pole of the spleen, a cyst is defined with clear smooth contours of 7.5 × 7.5 × 5 cm, uniform content, without septa or additional inclusions (Fig. 1). There were no signs of color Doppler mapping in the walls of the cyst. Conclusion: cyst of the spleen is not excluded.

Previously, spleen surgery had not performed.

During the surgery, a percutaneous puncture and MWA of the splenic cyst were performed under the control of ultrasound and X-ray fluoroscopy. Under intravenous anesthesia (Propofol 150 mg, Phentanylum 0.2 mg), an MWA catheter (MedWaves Incorporated, AveCure^®^) was inserted percutaneously into the cyst of the spleen's upper pole, accessing via the left mid-axillary line in the 9th intercostal space. In the epigastrium on the left anterior axillary line, a percutaneous puncture of the cyst of the upper pole of the spleen was made using a 18 gauge needle. After removal of the stylet, about 140 ml of a green turbid liquid was evacuated, which was sent for biological and cytological examination. Then, a radiopaque preparation (Urografin^®^ 76% - 20 ml + NaCl 0.9% 120 ml) was injected into the cyst. During X-ray, a neoformation of clear, even contours, and of uniform contrast was determined at the left subphrenic region. No contrast in the abdominal cavity or the vessels of the spleen was detected. MWA of the cysts was performed using a MWA catheter placed in the cavity at the center of the cyst, with the frequency of 902–928 Hz delivered during 5 minutes. The installed 18 G needle allowed the evacuation of the residual high temperature liquid during the MWA. Then, both the catheter and needle were removed.

Biological and cytological examination of the fluid obtained during the surgery revealed the presence of a cylindrical epithelium and the absence of abnormal cells.

The postoperative period was uneventful, and no analgesics required.

The abdominal ultrasound examination performed on day 2 post-surgery, did not reveal the residual cavity of the cyst in the region of the upper pole of the spleen.

The patient was discharged under the supervision of a general practitioner, with recommendations to abstain of physical activity for 3 months, and to perform ultrasound examinations every six months.

**Fig. 2:**
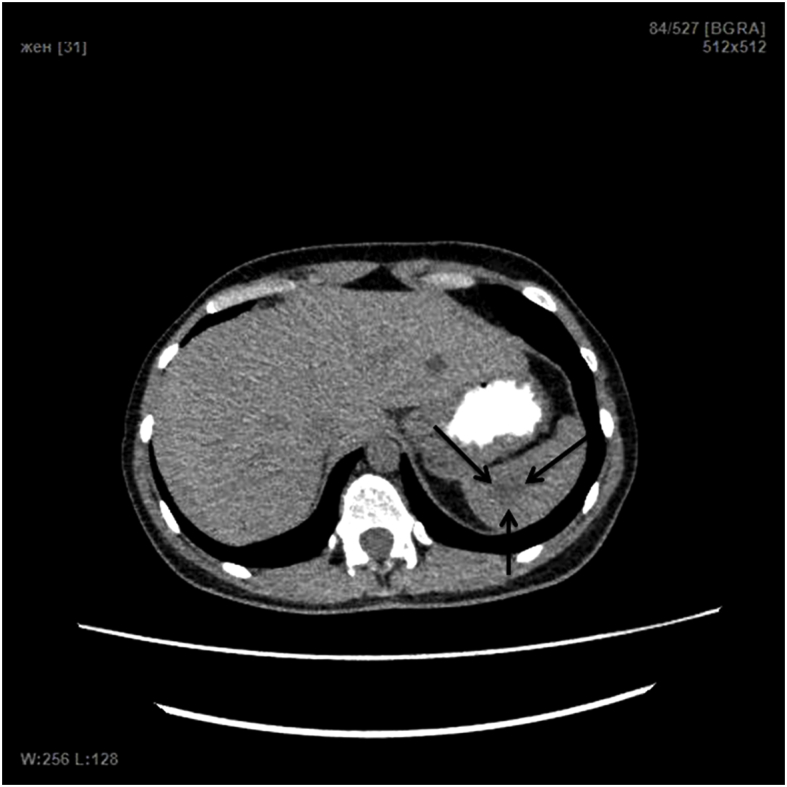
Four months post-surgery CT scan of the abdominal region on October 22, 2018. Note the residual cavity of the splenic cyst of 3 × 1.7 × 1.9 cm.

Four months post-surgery, on October 22, 2018, the patient underwent the control examination. There were no complaints reported. CT scan of the abdominal cavity in the region of the upper pole, has revealed the residual cavity of the cyst with dimensions of 3 × 1.7 × 1.9 cm (volume 4.8 ml) (Fig. 2).

## 3 Discussion

The spleen is an important internal organ involved in blood formation and ensuring the immunological status. Splenectomy, even performed using laparoscopic approaches, is a traumatic method requiring endotracheal anesthesia. The cost of this surgery reaches up to 250,000.00 rubles in Russia and $20,000.00 USD in the United States. The average bed-day is 12 ± 2.3, and the frequency of complications is up to 22.7% [7].

Sclerosis of the cyst using 96% ethyl alcohol often results in a relapse of the disease [4]. For instance, the 12.5% relapse, and the average bed-day of 18.5 was reported after the sclerosis of splenic cysts in 48 children [5,6].

Similar observation were reported in our clinic during sclerosis of the NSC in 50 patients [4,8]. To improve the method, we applied embolization of splenic artery that feeds the cystic formation of the spleen to induce its sclerosis using 96% ethanol. The outcomes were somewhat better, but after embolization of splenic artery, patients complained to severe pain in the left hypochondrium. The recurrence rate was 23.8%, and the average bed-day was 18 ± 2.3 [4].

Our novel MWA technique, to the best of our knowledge, was not described in the literature. This advanced approach of NSC treatment allows to carry out the procedure on an outpatient basis. MWA is less traumatic, does not require general anesthesia, and can be performed using intravenous or local anesthesia. There is no recurrence of the disease, and the duration of treatment is 1–2 bed-days.

The case described by us demonstrates the efficacy of MWA of NSC. The residual cavity of the cyst 4.8 ml, detected by abdominal CT. After the surgery, the patient had no pain, and the operation itself and the hospital stay were 2 days. There were no complications during and after the surgery.

MWA due to ultra-high-frequency (microwave) energy leads to rapid heating of the cyst contents and its walls up to 120 degrees. This contributes to necrosis and sclerosis of the cyst walls. By controlling the temperature and time, using the generator it is possible to perform a complete destruction of the walls of the cyst.

## 4 Conclusion

MWA is a promising approach in NSC treatment.

## Ethical approval

NA.

## Sources of funding

None.

## Conflicts of interest

None.

## Author contribution

Radmila Karpova performed the procedure, wrote the manuscript and is responsible for the information.

Artem Mishin and Samir Guseinov assisted in the operation, wrote the manuscript and are responsible for the information.

## Research registration unique identifying number (UIN)

NA.

## The trial registry number – ISRCTN

NA.

## Guarantor

Radmila Karpova, Artem Mishin, Samir Guseinov.

## Provenance and peer review

Not commissioned, externally peer reviewed.

## Patient consent

The patient provided her informed consent for the publication of her clinical details and any accompanying images about this case report.

## Appendix A Supplementary data

supp supp

## References

[1] Ingle S.B., Hinge (Ingle) C.R., Patrike S. (2014). Epithelial cysts of the spleen: a minireview. World J. Gastroenterol. WJG..

[2] Agha R.A., Borrelli M.R., Farwana R., Koshy K., Fowler A.J., Orgill D.P., SCARE Group (2018). The SCARE 2018 statement: updating consensus Surgical CAse REport (SCARE) guidelines. Int. J. Surg. Lond. Engl..

[3] (2015). Хирургическое Лечение Больных С Травматическими Повреждениями Селезенки. Ульяновский Медико-Биологический Журнал.

[4] Ширяев А.А., Мусаев Г.Х., Харнас С.С., Рябова А.В., Кондра♯ин С.А., Ло♯енов В.Б., Волкова А.И., Поминова Д.В., Жемерикин Г.А. (2013). Непаразитарные кисты селезенки. Методы хирургического лечения, Вестник Хирургической Гастроэнтерологии.

[5] Беляева О.А., Кондра♯ин С.А., Поляев Ю.А., Гарбузов Р.В., Мусаев Г.Х., Ширяев А.А. (2016). Комбинированные Навига⃛ионные Оперативные Вме♯ательства У Детей С Солитарными Кистами Селезенки. Российский Вестник Детской Хирургии Анестезиологии И Реаниматологии.

[6] López J.J., Lodwick D.L., Cooper J.N., Hogan M., King D., Minneci P.C. (2017). Sclerotherapy for splenic cysts in children. J. Surg. Res..

[7] Cai Y., Liu Z., Liu X. (2014). Laparoscopic versus open splenectomy for portal hypertension: a systematic review of comparative studies. Surg. Innov..

[8] Ширяев А.А. (2010). Диагностика и лечение непаразитарных кист селезенки.

